# Release of the Non-Steroidal Anti-Inflammatory Drug Flufenamic Acid by Multiparticulate Delivery Systems Promotes Adipogenic Differentiation of Adipose-Derived Stem Cells

**DOI:** 10.3390/ma13071550

**Published:** 2020-03-27

**Authors:** Andreea D. Lazăr, Sorina Dinescu, Mădălina G. Albu-Kaya, Sami Gharbia, Anca Hermenean, Marieta Costache

**Affiliations:** 1 Department of Biochemistry and Molecular Biology, University of Bucharest, 050095 Bucharest, Romania; andreea.lazar@bio.unibuc.ro (A.D.L.); marieta.costache@bio.unibuc.ro (M.C.); 2 Research Institute of the University of Bucharest, 050663 Bucharest, Romania; 3 Department of Collagen Research, Division of Leather and Footwear Research Institute, The National Research & Development Institute for Textiles and Leather, 031215 Bucharest, Romania; albu_mada@yahoo.com; 4 Institute of Life Sciences, Vasile Goldis Western University of Arad, 86 Rebreanu, 310414 Arad, Romania; samihgh@hotmail.com (S.G.); anca.hermenean@gmail.com (A.H.)

**Keywords:** adipose tissue engineering, multiparticulate drug delivery systems, flufenamic acid, adipose-derived stem cells, adipogenic differentiation, PPARγ2, perilipin

## Abstract

Engineered tissue-like structures often instigate an inflammatory response in the host that can inhibit wound healing and ultimately lead to the rejection of the implant. In our previous study, we have characterized the properties and biocompatibility of novel multiparticulate drug delivery systems (MDDS), based on collagen matrix with gradual release of anti-inflammatory drug flufenamic acid, we evaluated their anti-inflammatory potential and demonstrated their efficiency against burns and soft tissue lesions. In addition to these results, FA was previously described as a stimulant for adipogenesis, therefore we hypothesized that MDDS might also be appropriate for adipose tissue engineering. After the cell-scaffold constructs were obtained, cell morphology, adhesion and spreading on the systems were highlighted by scanning electron microscopy, immunostaining and confocal microscopy. The effect of FA-enriched materials on adipogenesis was evaluated at gene and protein level, by RT-qPCR, confocal microscopy and immunohistochemistry. Our current work indicates that flufenamic acid plays a beneficial role in adipocyte differentiation, with a direct effect upon the gene and protein expression of important early and late markers of adipogenesis, such as PPARγ2 and perilipin.

## 1. Introduction

The field of tissue engineering (TE) combines principles of engineering with notions from natural sciences in order to develop complex structures capable of mimicking natural tissue, in both aspect and function, fit for the purpose of regeneration [1]. Adipose tissue engineering (ATE) belongs to this interdisciplinary field and it aims to obtain a proper substitute for damaged adipose tissue, resulted for example, from tumor resection, burns or other types of wounds. In vitro an ATE construct can be generated by seeding a biomaterial, made out of natural/synthetic compounds or a combination of the two, with cells capable of differentiating in mature adipocytes in the presence of a proper differentiation media (adipogenic inducers). Once implanted at the lesion site, the construct should integrate with the normal tissue through in vivo remodeling [2,3].

The best choice for cellular component would seem to be human adipose-derived stem cells (hASCs), hypo-immunogenic cells suitable for designing biocompatible tissue constructs, which can be easily harvested in abundance from autologous fat tissue through minimal invasive procedures [4,5]. hASCs’ self-renewal ability is similar to bone marrow or umbilical cord stem cells, and they are capable of differentiating into cells of mesodermal or ectodermal lineages under the influence of a specific inducing microenvironment [6,7,8,9,10,11]. Moreover, hASCs release multiple growth factors that facilitate tissue regeneration, such as vascular endothelial growth factor (VEGF), hepatocyte growth factor (HGF), fibroblast growth factor-2 (FGF-2), transforming growth factor (TGF), platelet-derived growth factor-b (PDGFB), angiopoietin-1 (Ang-1), Ang-2, stromal cell-derived factor-1 (SDF-1) and osteonectin [12]. Preclinical studies on the use of hASCs in vitro and in vivo have been performed, and their efficacy has been established in clinical trials [13,14,15].

The differentiation of hASCs involves a complex series of changes in the cellular gene and protein expression patterns. In vitro differentiation is characterized by growth arrest, the induction and expression of multiple adipogenic genes, and ultimately, triglyceride accumulation [16,17]. One of the key regulators of the process is peroxisome proliferating activated receptor gamma-2 (PPARγ2), a ligand-activated transcription factor that belongs to the nuclear receptor superfamily, which is selectively expressed in adipocytes and induced early (initial 24–48 h) during the course of differentiation [18,19]. After hormonal induction, PPARγ2 level significantly increases, thus initiating adipogenesis, and then gradually decreases as the process evolves. PPARγ2 also plays a role in maintenance of the differentiated state. If silenced, already differentiated 3T3-L1 preadipocytes express specific late markers, such as perilipin, at a lower level and the accumulated fat droplets diminish, thus dedifferentiating [20]. Perilipin is considered a late marker of adipogenesis because it coats the intracellular lipid droplets, which only form in the terminal stage of the process, acting as a protective barrier that restricts the access of cytosolic lipases. Its expression is mainly regulated by PPARγ2 [21,22].

In order to provide the cells with an in vivo-like structure, tridimensional (3D) scaffolds that resemble the natural extracellular matrix (ECM) in architecture and composition should be used, because they allow the cells to maintain their conformation, communicate and interact with one another, exert their native functions and better adhere to the material, thus facilitating the formation of a proper tissue construct [23]. Due to their high biocompatibility and similarity to the ECM, natural polymers are widely used for ATE purposes [24,25]. Collagen is prevalent in native adipose ECM, has low antigenicity, low inflammatory properties and good biocompatibility, promoting cell attachment and favorable adipose outcome [26,27,28]. Even more, it is approved for use in humans by the US Food and Drug Administration (FDA) [24]. However, pure collagen has a fast degradation rate in vivo; therefore, to enhance its mechanical strength and durability it needs to be cross-linked with other polymers (such as dextran) or fixatives (for example glutaraldehyde) [29].

To facilitate the future integration of the ATE construct into the lesion site, various drugs, natural plant extracts or growth factors can be incorporated into the biomaterials [16,30]. For example, Kimura et al. combined collagen sponges with gelatin microspheres containing basic fibroblast growth factor (bFGF) and implanted them in nude mice. Within 6 weeks, adipose tissue developed in the constructs and the extent of fat tissue formation was positively influenced by the bFGF concentration, presumably by promoting angiogenesis [31]. Nonsteroidal anti-inflammatory drugs (NSAIDs), such as flufenamic acid (FA), modulate the immune response of the host, and diminish the excess inflammation that could occur as a result of implantation, a potential cause for construct rejection. NSAIDs are commonly used in the clinic for their anti-inflammatory, antipyretic and analgesic activity. Therapeutic actions are attributed to inhibition of cyclooxygenases (COX-1 and COX-2), enzymes that catalyze the first steps in conversion of arachidonic acid (AA) to prostaglandins (PGs) [32]. While the COX enzymes constitute major NSAID targets, there are also suggestions that some NSAIDs effects are mediated by PPAR members. High doses of certain NSAIDs concentrations can modulate PPARγ activation in vitro [33,34]. FA is a good candidate for ATE applications because in addition to its proven conventional role, a previous study reported that FA facilitates differentiation into fat tissue, acting as an adjuvant to the process in a similar, though less potent, manner to another class member, namely indomethacin, regularly used as a component in the adipocyte differentiation media [33,35]. As such, FA may contribute to the activation of PPARγ2, the main inducer of adipogenesis, in the first 1–2 days of differentiation [36].

To reduce the initial burst release effect and to provide a controlled drug release over time, our group previously proposed novel multiparticulate drug delivery systems (MDDS) for FA, based on collagen composite matrices with gelatin-alginate-carboxymethylcellulose microcapsules for drug encapsulation, that allowed the drug to be rapidly discharged in the first 60 min and then gradually released over the course of 48 h. Out of all the tested MDDS, the ones with the highest amount of FA, specifically M2 and M4 (systems with 30% FA microcapsules in their structure and additional FA in free form incorporated in the composite gel of M4) obtained the best results, the released FA percentage reaching 85.3% for M2 and 95.01% for M4. Moreover, these natural polymeric systems showed good biocompatibility, having a positive influence on hASCs’ viability and proliferation, at the same time allowing the gradual degradation of the collagen support and the slow release of the drug [37]. Such kinetic profiles with drug biphasic release may facilitate de activation of PPARγ2, the main adipogenic inducer, whose expression is induced early (24–48 h) in the differentiation process [36], therefore M2 and M4 were chosen for further evaluation.

In the present study, we aimed to evaluate the potential of MDDS to sustain adipogenesis in vitro and the effects of the gradual release of FA over 48 h on adipogenic differentiation. Our results showed the successful formation of the cell-scaffold constructs (confocal microscopy and scanning electron microscopy), as well as the successful differentiation of hASCs (scanning electron microscopy), with better accumulation of intracellular lipid droplets (Oil Red O staining) and increased PPARγ2 and perilipin expression levels (RT-qPCR, immunohistochemistry and immunofluorescence coupled with confocal microscopy) in MDDS compared to a collagen composite matrix without FA. Overall, our study indicates FA as an adjuvant to adipocyte differentiation and provides valuable clues for its potential use in ATE applications.

## 2. Materials and Methods 

A primary culture of hASCs was obtained from lipoaspirate, which was collected after the informed consent of the patient, following a well-established protocol [38,39]. All cell-based experiments were approved by the Ethics Committee of the University of Bucharest and were in compliance with the Declaration of Helsinki. Cells were cultured in Dulbecco’s Modified Eagle’s Medium (DMEM) supplemented with 10% fetal bovine serum (FBS), 1% antibiotic antimycotic solution (Sigma-Aldrich, Darmstadt, Germany) and maintained in standard conditions (37 °C, 5% CO_2_, humidity). Cell media was changed every 2–3 days and upon reaching ~80% confluence, they were passaged with trypsin solution (Sigma-Aldrich).

Systems M2 and M4 were prepared as described in [37]. The samples were identical in terms of collagen, dextran and crosslinking agent (glutaraldehyde) concentration, with a difference in FA concentration: M2 incorporated 30 g microcapsules and 70 g composite gel without FA, while M4 consisted of 30 g microcapsules and 70 g composite gel with FA. Composite gel (COL) without FA or microcapsules was used as control (Table 1). hASCs at passage 5 were seeded on the scaffolds, at a density of 2 × 10^5^ cells/cm^2^, and allowed to distribute inside the 3D materials over 24 h.

After 24 h of culture, adhered cells on the 3D systems were fixed with a 4% paraformaldehyde solution for 20 min, permeabilized with 2% bovine serum albumin (BSA) solution with 0.1% Triton X100 for 1 h, stained with fluorescein isothiocyanate (FITC)-conjugated phalloidin for 20 min and 4, 6-diamidino-2-phenylindole (DAPI) for 5 min. All solutions and staining agents were purchased from Sigma-Aldrich. Cells were visualized by confocal microscopy with a Carl Zeiss LSM 710 Confocal Microscope System (Zeiss, Germany).

To further asses the morphology, adhesion and distribution of hASCs in the 3D systems, the samples were also captured with scanning electron microscopy (SEM), after 7 days of culture. The constructs were mounted on conductive aluminum pin stub specimen and metallized with gold using a sputter coater Agar with a layer of 3 nm thickness/deposition for 3 times. Examination and image analysis were conducted on a Quanta 250 microscope (FEI, Frankfurt am Main, Germany).

To induce adipogenic differentiation of hASCs, 24 h after seeding, the culture media was changed to a commercially available cocktail of adipogenic inducers (StemPro Adipogenesis Differentiation Kit from Thermo Fischer Scientific, Waltham, MA, USA), and the cell-scaffold constructs were exposed to this differentiation media, changed every three days, over a period of 21 days. Evolution of the adipogenic process was monitored at gene expression and protein level by analyzing the expression of early marker PPARγ2 and late marker perilipin. Intracellular lipids were also detected with Oil Red O staining. After 14 and 21 days of differentiation, cell media was removed, the constructs were washed with phosphate buffered saline (PBS) solution and fixed overnight with Immunofix (Bio-Optica, Milano, Italy). The samples were cryopreserved in liquid nitrogen, sectioned at the RM2125-RT microtome and placed on X-tra® Slides (Leica Biosystems, Nussloch, Germany). Intracellular lipid droplets were stained with Oil Red O solution purchased from Sigma-Aldrich (5 mg/mL in 60% isopropanol diluted 3:2 with distilled water). Nuclei were marked with hematoxylin and the samples were dehydrated through ascending alcohols (Unyhol and Unyhol Plus solutions, Bio-Optica), cleared (BioClear from Bio-Optica, Italy) and mounted (CV Mount solution, 14046430011, Leica Biosystems, Nussloch, Germany). Images were taken with the digital camera (Olympus XC30) of a Bx43 microscope (Olympus, Tokyo, Japan). Cell morphology and distribution of differentiated hASCs in the constructs were assessed by SEM, following the previously described protocol.

Gene expression of PPARγ2 and perilipin was evaluated after 7, 14 and 21 days of adipogenic differentiation. Cells were isolated by digesting the materials with collagenase solution (0.2% type I collagenase, 0.2% BSA, 3 mM CaCl_2_, 1% antibiotic), 2-4 h at 37 °C. Total RNA was extracted using TRIzol Reagent (Thermo Fisher Scientific, Waltham, MA, USA) in accordance with the manufacturer’s instruction, and assessed for concentration and purity on a NanoDrop™ 8000 Spectrophotometer (Thermo Fisher Scientific, Waltham, MA, USA), and for integrity on the BioAnalyzer 2100 (Agilent Technologies, Waldbronn, Germany). Complementary DNA was synthetized using iScript cDNA Synthesis kit (BioRad, Hercules, CA, USA) from 1000 ng RNA/reaction, and amplified through PCR on Veriti 96 Well Thermal Cycler from Applied Biosystems (Waltham, MA, USA). Adipogenic markers’ expression was evaluated by Real-Time PCR, performed on LightCycler 2.0 carrousel-based system with FastStart DNA Master SYBR Green I Kit (Roche, Rotkreuz, Switzerland). All samples were evaluated in triplicate and normalized to the expression of TATA-binding protein (TBP) house-keeping gene. The specific primer sequences used for gene expression assessment are presented in Table 2.

One-way ANOVA method followed by Bonferroni’s multiple comparison test was performed in order to statistically analyze the data. The results were expressed as a mean ± S.D. using GraphPad Prism Software, version 6 for Windows (GraphPad Software, San Diego, CA, USA).

PPARγ2 and perilipin protein expression were evaluated by immunohistochemistry after 7 and 14 days, respectively 7 and 21 days of adipogenesis, using Novolink Polymer Detection Systems Novocastra (RE7280-K, Leica Biosystems) according to the manufacturer’s instructions. The constructs were fixed with Immunofix, embedded in paraffin and sectioned at the microtome (Leica Biosystems). The paraffin sections were dewaxed with Dewax solution (AR9222, Leica Biosystems) and rehydrated in different alcohol concentrations (100%, 95%, 70%), then they were stained overnight with specific primary antibodies (Santa Cruz Biotechnology, Heidelberg, Germany) for PPARγ2 (sc-22022) and perilipin (sc-67164). Novolink polymer highlighted the protein levels of PPARγ2 and perilipin. Images were taken with the digital camera of the Olympus Bx 43 instrument. Furthermore, the markers’ protein expression was also evaluated by immunofluorescence and confocal microscopy. The cells inside the constructs were fixed, permeabilized and stained with primary antibodies as previously described. After that, the samples were washed with PBS and incubated for 1 h with specific secondary antibodies coupled with fluorophores, FITC-conjugated for PPARγ2 (sc-2777), and tetramethyl rhodamine isothiocyanate (TRITC)-conjugated for perilipin (sc-2091), purchased from Santa Cruz Biotechnology. Nuclei were stained for 5 minutes with DAPI (Sigma-Aldrich). Afterwards, the constructs were visualized at the confocal microscope.

## 3. Results

We tested two MDDS (M2 and M4), both of them with the same amount of FA microcapsules (30%), but one of them enriched with FA in the composite gel (M4), against a simple composite gel without FA or microcapsules (COL), as potential candidates for adipose tissue engineering. After seeding with hASCs, we firstly assessed the morphology, adhesion and distribution of the cells in the 3D materials by immunostaining coupled with confocal microscopy, followed by SEM.

### 3.1. Assessment of the Tridimensional Cell-Scaffold Constructs Formation

**Figure 1:**
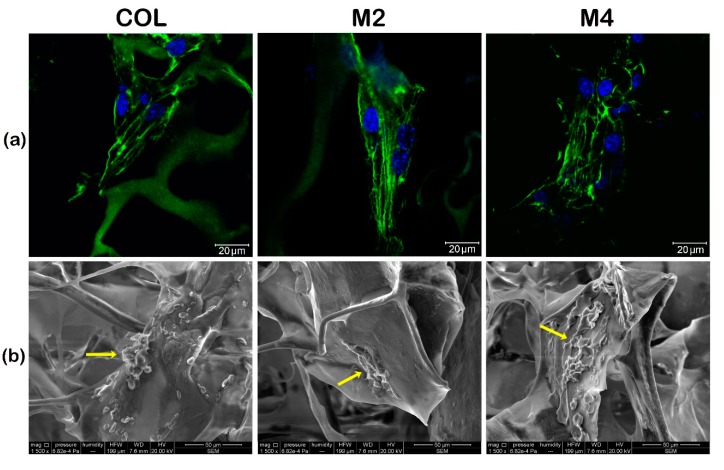
Evaluation of undifferentiated hASCs morphology, adhesion and distribution in the 3D materials by: (**a**) confocal microscopy and fluorescent staining; (**b**) SEM (yellow arrows pinpoint the cells attached to the MDDS).

The successful formation of the cell-scaffold constructs was determined by immunofluorescence staining of actin filaments 24 h after seeding and confocal microscopy, which showed the developed cells’ cytoskeleton in contact with the materials (Figure 1a).

On all constructs, the actin developed into filaments, corresponding to a spindle-like shape, highlighting the ability of the 3D collagen-based systems to support hASCs’ adhesion and spreading. This, in addition to MTT, LDH and Live/Dead assays previously done [37], further confirms the biocompatibility of the tested MDDS.

To further evaluate cell morphology, adhesion to the materials and dissemination into the 3D structure, we utilized SEM and observed that after 7 days of culture, hASCs displayed an elongated phenotype, were attached to the scaffolds and evenly spread into their structure, confirming the successful formation of cell-scaffold constructs (Figure 1b).

### 3.2. Evaluation of Flufenamic Acid Effect on Adipogenic Differentiation of Human Adipose-Derived Stem Cells 

The effect of FA incorporated in the materials on the evolution of adipogenesis was monitored for 21 days and evaluated by measuring the gene expression and protein level of PPARγ2 and perilipin, as well as marking intracellular lipid droplets with Oil Red O for an initial assessment.

#### 3.2.1. Evaluation of Intracellular Lipid Accumulation

**Figure 2:**
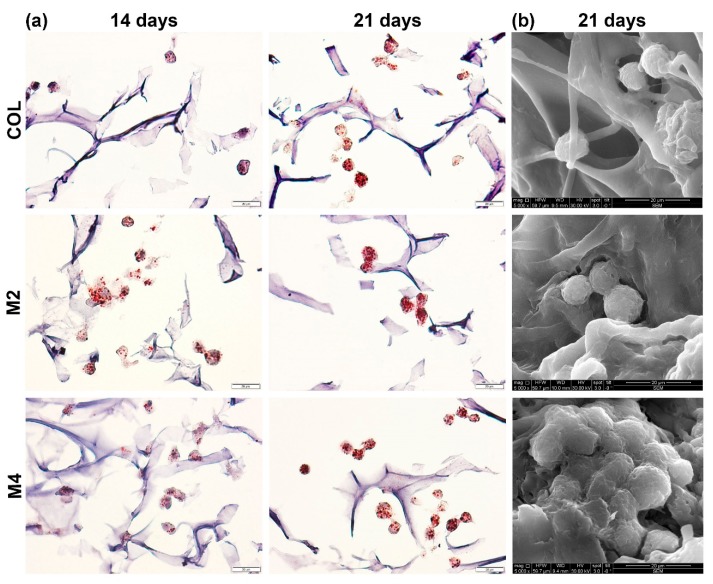
(**a**) Evaluation of intracellular lipid accumulation after 14 and 21 days of hASCs adipogenic differentiation using Oil Red O staining; (**b**) Rounded phenotype of differentiated cells visualized by SEM after 21 days of adipogenic differentiation.

Histological staining with Oil Red O revealed the lipid droplets stored in the cellular compartment, indicating that hASCs underwent adipogenesis and evolved into adipocytes. A higher amount of intracellular lipids (marked in red) was observed for the cells seeded on the MDDS, than the pure composite gel (COL) after 14 and 21 days of differentiation (Figure 2a).

No lipid droplets were observed at 7 days as the process of differentiation was still in the early stages, but after 14 days of adipogenesis neutral lipids could be distinguished in the cells seeded on the three tested materials. A better proportion of lipid droplets was found on the materials who had FA in their structure, suggesting a positive role on adipogenic differentiation for this anti-inflammatory drug. The small amount of FA merged into the composite gel of M4 seemed to further enhance the process, as a higher amount of lipids can be seen on this MDDS, compared to M2 who had only FA microcapsules. Evaluation at 21 days of differentiation confirmed the benefic effect of FA, suggested after 14 days, on the evolution of adipogenesis. A stronger accumulation of voluminous intracellular lipids droplets could be observed in the MDDS, when compared to COL control. In order to visualize the differentiated cell morphology of hASCs and dissemination into the 3D structure, we utilized SEM and observed that after 21 days of culture, hASCs displayed a rounded phenotype and were evenly spread into their structure, confirming the successful differentiation of cells seeded on the constructs (Figure 2b).

#### 3.2.2. Gene Expression Evaluation of Early and Late Markers of Adipogenesis 

**Figure 3:**
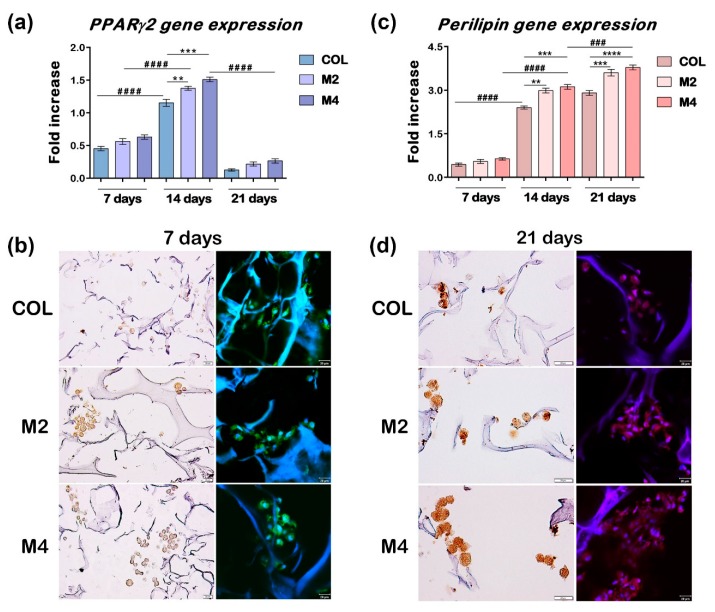
Evaluation of early and late markers of adipogenesis: (**a**) PPARγ2 gene expression level at 7, 14 and 21 days after induction of adipogenesis; (**b**) PPARγ2 protein expression visualized by immunohistochemistry and immunofluorescence coupled with confocal microscopy after 7 days of adipogenic differentiation; nuclei are stained in blue (DAPI) and PPARγ2 is stained in green (FITC); (**c**) Perilipin gene expression level at 7, 14 and 21 days of adipogenesis; (**d**) Immunohistochemistry and immunofluorescent staining of perilipin protein expression after 21 days of adipogenic differentiation. Nuclei are stained with DAPI (blue); perilipin is red (TRITC). All samples were evaluated in triplicate and the results are expressed as a mean ± S.D. Statistical significant differences are *p* < 0.01 (**); *p* < 0.001 (***); *p* < 0.0001 (****). Highlighted with * are the statistical differences between different materials at the same time, and with # are the statistical differences between the same material but at different times.

The progression of adipogenesis was monitored at gene expression level by measuring two important markers of differentiation, namely *PPARγ2* and *perilipin*, at 7, 14 and 21 days from the initial start of the process (Figure 3a,c).

For all tested composites, a statistically significant increase in *PPARγ2* expression was observed from 7 to 14 days of differentiation (*p* < 0.0001), along with a significant decrease from 14 to 21 days (*p* < 0.0001), as expected for this early marker of adipogenesis. After 7 days, no statistical difference was noticed for *PPARγ2* expression between the tested materials, but after 14 days there was a significant difference between the COL control and MDDS, suggesting the positive effect of FA on adipogenesis. Even though no statistical difference was detected between M2 and M4, a distinction between them was observed when compared to the composite gel control, with a more significant difference for M4 (*p* < 0.001), than M2 (*p* < 0.01), indicating the effect of the free FA contained in the composite gel of M4. After 21 days of differentiation, the expression of *PPARγ2* decreased considerably, but remained at approximately the same level on all three composites (Figure 3a).

*Perilipin* expression evaluation revealed a continuous significant increase over the course of 21 days, with *p* < 0.0001 from 7 to 14 days, *p* < 0.001 on M4 from 14 to 21 days, and with *p* < 0.01 for COL and M2 from 14 to 21 days, confirming the evolution of differentiation and the cumulative benefic effect of FA. Similar to *PPARγ2* assessment, no statistical difference was detected between the expressions of perilipin on the three composites after 7 days of differentiation. However, after 14 days, MDDS registered a much higher expression of *perilipin* compared to COL, with *p* < 0.01 for M2 and *p* < 0.001 for M4, respectively. This difference increased after 21 days of adipogenesis, with *p* < 0.001 for M2 and *p* < 0.0001 for M4, suggesting again the positive influence of FA on the evolution of adipocyte development (Figure 3c).

#### 3.2.3. Protein Expression Evaluation of Early and Late Markers of Adipogenesis 

The protein expression of PPARγ2 and perilipin was evaluated by immunohistochemistry and immunofluorescence coupled with confocal microscopy, in order to confirm the results obtained at gene level. Considering the fact that PPARγ2 has its expression peak during the beginning of the process, the assessment was done at 7 and 14 days post-initiation of adipogenesis. Because perilipin is expressed later in the process, the protein level detection was performed at 7 and 21 days of adipogenic differentiation. A significant rise in protein level was detected for PPARγ2 after 7 days (Figure 3b), and for perilipin after 21 days (Figure 3d), on all the tested materials, suggesting the progression of the adipogenic process. A higher protein expression could be assessed on the composites with FA, compared to the control. The addition of FA in the composition of the collagen-based materials seemed to have a benefic effect on the expression level of these two markers of adipogenesis, confirming the gene expression results. A slight positive difference could be detected on M4, who also had free FA in its composition, compared to M2 who only had encapsulated FA (Figure 3b,d).

## 4. Discussion

NSAIDs display anti-inflammatory, antipyretic and analgesic properties [32] and fall into many chemically distinct classes, including oxicams (piroxicam), indole derivatives (indomethacin), acetic acid derivatives (diclofenac), aminoacyl carboxylic acids (FA), arylpropionic acid (ibuprofen and fenoprofen) and acid acetylsalicylic (aspirin) [34,40]. While the molecular basis for the therapeutic actions of NSAIDs is believed to be their ability to inhibit COX activity and thereby block the production of PGs [32], some of these compounds can modulate PPARs [33,41]. This finding raises the possibility that such off target NSAID effects contribute to the spectrum of actions of these drugs [34].

Several studies have focused on the effects of different NSAIDs on preadipocyte cell lines or mesenchymal stem cells (MSCs), including a series of biological behaviors such as adhesion, proliferation, and differentiation [33,34,42,43], albeit the effect differs with the change in drug and concentration. In this study, we set out to evaluate the potential to promote adipogenesis of novel MDDS improved with FA and determine whether or not FA influences this process. Initial assessment of cell-scaffold constructs by SEM and confocal microscopy confirmed their formation, while the following experiments revealed the successful adipogenic differentiation of hASCs seeded on MDDS, as judged by SEM analysis and Oil Red O staining, with multiple, more pronounced intracellular lipid droplets compared to the composite gel without FA, indicating that FA positively affects the differentiation of hASCs.

Little evidence of the promoting effect of FA on adipogenic differentiation has been published, except for a report from Lehmann and colleagues that showed that FA is a PPARγ ligand and micromolar concentrations (100 μM) activate this transcription factor, inducing adipogenesis in a similar manner (although less efficiently) to indomethacin – a fellow NSAID and COX inhibitor, frequently included in the commercial mixture used to promote in vitro differentiation of various preadipocyte cell lines/stem cells [33]. In this regard, we aimed at evaluating the efficiency of FA as a potential inducer of PPARγ2 activation. It is well known in literature that PPARγ2 expression is induced in the first 24–48 h from the initiation of adipogenesis by specific signals [36], therefore our group intentionally designed two systems (M2- with encapsulated FA, and M4- with FA not only in microcapsules, but also in the composite gel), with gradual release of the drug over 48 h, the same amount of time necessary for PPARγ2 activation. We aimed not at evaluating the effect of FA over the entire differentiation process, instead focusing on the role of this NSAID as a potential promoter of PPARγ2 activation. Although we did not study in detail the molecular mechanisms behind the benefic effect of FA on adipogenesis, our results concerning the expression of PPARγ2 at gene and protein level are in accordance with previous analyses. RT-qPCR, immunohistochemistry evaluation and confocal microscopy assessment revealed significant differences between M2 (encapsulated FA), M4 (free FA and microcapsules) and the composite gel without the drug. As expected, statistically higher *PPARγ2* expression was registered on MDDS when compared to the control, with *p* < 0.001 for M4 and *p* < 0.01 for M2, suggesting that even the slight amount of FA dispersed in the matrix composition contributed to adipogenesis progression. Protein analysis confirmed the gene expression results, with stronger coloration and fluorescent signal being present in MDDS, compared to COL.

To our knowledge, this is the first report that proves the positive upregulation of perilipin, a terminal marker of adipocyte differentiation, in the presence of FA. Gene expression evaluation revealed a continuous significant increase over the course of differentiation, confirming the evolution of the process and the cumulative benefic effect of FA, with statistically significant increased *perilipin* expression on the M2 and M4 systems, compared to control. Initially, no difference between the composites could be detected, but eventually MDDS registered a much higher expression of *perilipin* compared to COL, with *p* < 0.01 for M2 and *p* < 0.001 for M4, ultimately reaching *p* < 0.001 for M2 and *p* < 0.0001 for M4 at the end of the process. This important difference between the control material without FA and MDDS indicated the positive influence of FA on the evolution of adipocyte development. Again, the results obtained at gene expression level were confirmed by protein analysis. Presence of FA seemed to have a benefic effect on the protein levels of perilipin; stronger precipitation and pronounced fluorescence could be observed on the composites with this particular NSAID, compared to the control. Even more, M4 (additional FA in its composition) registered the strongest protein expression, suggesting that adipogenic differentiation is better promoted by higher concentrations of FA. This finding is in accordance to what Lehmann et al. observed [33], but in contrast to what Liu et al. reported for bone tissue engineering, where small concentrations of FA promoted osteogenesis, while high FA concentrations hampered the process’ development [43].

In addition to our current report which promotes MDDS as good biomaterials for future adipose tissue constructs, in a previous study we demonstrated the biocompatibility and anti-inflammatory efficiency of M2 and M4 systems and validated MDDS for drug delivery in wound healing applications [37]. MDDS are polymeric matrices based on collagen-dextran and embedded microcapsules of gelatin-carboxymethylcellulose-alginate that enable the gradual delivery of FA, thus reducing the initial burst release effect and assuring the even distribution of the drug over a determined period of time. Their biocompatibility was investigated, followed by evaluation of the release mechanism, degradation rate, absorption capacity and efficiency against in vitro inflammation modelling in vivo behavior. All studied MDDS displayed good biocompatibility, in particular the matrices with 30% FA microcapsules (M2 and M4). MDDS showed good absorbent properties and degraded gradually, thus facilitating the gradual release of the therapeutic agent over the course of 48 hours (a time period proportional with the one required for initial activation of PPARγ2). Systems M2 and M4 also registered the highest anti-inflammatory efficiency, most likely attributed to the gradual release of the drug from the microcapsules. In animal models, the biopolymers associated with FA accelerated the healing process, improving epithelial regeneration and insuring minimal scarring, the best results being obtained for the systems with the highest concentration of FA (M2 and M4). Also, no secondary systemic or topic effects were associated with MDDS treatment, as compared to the untreated control group which developed impaired topical inflammation and had a prolonged healing period.

Our current results promote novel MDDS, in particular the one with the highest concentration of FA (M4, with both microcapsules and free form FA), seeded with hASCs, as potential candidates for ATE applications. FA acts not only as an anti-inflammatory drug [37], but also as an adjuvant for adipogenic differentiation. This NSAID can be easily obtained, is not expensive and is already approved by FDA to treat several diseases, such as rheumatic arthritis, which constitute significant advantages for its clinical use [43,44]. Therefore, implication of FA in future ATE applications could be considered a safe and economical choice.

## 5. Conclusions

Cell-scaffold constructs were successfully obtained and visualized through SEM, immunostaining and confocal microscopy. MDDS supported the differentiation of hASCs as shown by SEM analysis. Intracellular accumulation of fat droplets, confirmed by Oil Red O staining, was higher in MDDS compared to control, suggesting the adjuvant role of FA. Significantly increased gene and protein expression levels for PPARγ2, an early marker of differentiation, and late marker perilipin, were registered for MDDS, compared to control, reinforcing the benefic effect of FA on adipogenesis. Slightly better results were obtained for M4 (FA in free form and microcapsules), compared to M2 (only encapsulated FA), but overall, our results showed that novel MDDS can sustain adipogenesis and that the gradual release of FA incorporated into the MDDS over the course of 48 h has a benefic effect on the evolution of adipogenic differentiation, being proportional with the time frame required for initial PPARγ2 activation. Nonetheless, further studies are needed to confirm these promising results and establish FA not only as an anti-inflammatory drug, but also as an adjuvant in potential ATE applications.

## Figures and Tables

**Table 1:** The Composition of the Studied Materials.

Systems	Composite Gel %				Microcapsules with FA %
	Collagen	Dextran ^1^	Glutaraldehyde ^1^	FA ^1^	
COL	0.8	1.2	0.006	-	-
M2	0.8	1.2	0.006	-	30
M4	0.8	1.2	0.006	0.5	30

^1^ Reported with respect to collagen, which means 0.96 g of dextran, 0.0048 g glutaraldehyde and 0.4 g FA in 100 mL of gel.

**Table 2:** The Specific Primer Sequences used for Gene Expression Assessment.

Gene	Forward Primer	Reverse Primer
PPARγ2	5’-TTACACAATGCTGGCCTCCTT-3’	5’-AGGCTTTCGCAGGCTCTTTAG-3’
perilipin	5’-ATGCTTCCAGAAGACCTACA-3’	5’-CAGCTCAGAAGCAATCTTTT-3’
TBP	5’-AGGCATCTGTCTTTGCACAC-3’	5’-GGGTCAGTCCAGTGCCATAA-3’
